# Endoscopic Surveillance for Colorectal Cancer in Pediatric Ulcerative Colitis: A Survey Among Dutch Pediatric Gastroenterologists

**DOI:** 10.1097/PG9.0000000000000341

**Published:** 2023-07-17

**Authors:** Jasmijn Z. Jagt, Daniëlle A. van Schie, Marc A. Benninga, Patrick F. van Rheenen, Nanne K. H. de Boer, Tim G. J. de Meij

**Affiliations:** From the * Department of Pediatric Gastroenterology, Emma Children’s Hospital, Amsterdam University Medical Centre, VU University Amsterdam, Amsterdam, The Netherlands; † Amsterdam UMC, VU University Amsterdam, Pediatric Gastroenterology, Amsterdam Gastroenterology Endocrinology Metabolism (AGEM), Amsterdam, The Netherlands; ‡ Faculty of Medicine, Amsterdam University Medical Centre, VU University Amsterdam, Amsterdam, The Netherlands; § Department of Pediatric Gastroenterology and Nutrition, Emma Children’s Hospital, Amsterdam University Medical Centers, University of Amsterdam, Amsterdam, The Netherlands; ∥ Department of Pediatric Gastroenterology, Hepatology and Nutrition, University of Groningen, University Medical Centre Groningen, Groningen, The Netherlands; ¶ Department of Gastroenterology and Hepatology, Amsterdam Gastroenterology Endocrinology Metabolism (AGEM) Research Institute, Amsterdam University Medical Centre, VU University Amsterdam, Amsterdam, The Netherlands.

**Keywords:** ulcerative colitis, pediatrics, surveillance, colorectal cancer, dysplasia

## Abstract

**Objectives:**

This study aimed to evaluate the current clinical practice of Dutch pediatric gastroenterologists regarding the surveillance for colorectal dysplasia and cancer in pediatric ulcerative colitis (UC), including adherence to guidelines, the initiation and interval of surveillance and applied endoscopy techniques.

**Methods:**

A clinical vignette-based survey was distributed among all 47 pediatric gastroenterologists who are registered and working in the Netherlands.

**Results:**

Thirty-three pediatric gastroenterologists treating children with UC, completed the questionnaire (response rate 70%). Of these respondents, 23 (70%) do conduct endoscopic surveillance in their UC patients. Adherence to any of the available guidelines was reported by 82% of respondents. Twenty-four of 31 respondents (77%) indicated the need for development of a new guideline. Profound variation was witnessed concerning the initiation and interval of surveillance, and risk factors taken into consideration, such as disease extent and concomitant diagnosis of primary sclerosing cholangitis (PSC). The available national and European guidelines recommend the use of chromoendoscopy in the performance of surveillance. This technique was conducted by 8% of respondents, whereas 50% conducted conventional endoscopy with random biopsies.

**Conclusions:**

The heterogeneity in surveillance practices underlines the need for consistency among the guidelines, explicitly stated by 77% of the respondents. For this, future research on surveillance in pediatric UC is warranted, focusing on the risk of UC-associated colorectal cancer related to risk factors and optimal endoscopy techniques.

**What Is Known**

- Recommendations on surveillance for colorectal cancer (CRC) in pediatric ulcerative colitis (UC) differ among European and national guidelines.
- Data on surveillance in pediatric UC regarding optimal interval and applied colonoscopy techniques are lacking.

**What is New**

- Heterogeneity was observed in surveillance practices of Dutch pediatric gastroenterologists for pediatric UC, particularly in applied endoscopy techniques and on the initiation and interval of surveillance and related risk factors.
- Among respondents, 77% indicated the need for the development of new guidelines, incorporating risk factors and disease extent.
- Future research using population-based cancer registries is needed to investigate the risk of dysplasia and CRC in childhood-onset UC, which should form the basis for developing new guidelines on surveillance practices in pediatric UC.

## INTRODUCTION

Childhood-onset ulcerative colitis (UC) has been described to be associated with a 33-fold higher risk of developing colorectal cancer (CRC) later in life compared with matched general population reference individuals (1,2). However, only a few cases of dysplasia and CRC in UC patients before 18 years old have been reported so far (3–6). According to the European Society of Pediatric Gastroenterology, Hepatology and Nutrition (ESPGHAN)/European Crohn’s and Colitis Organization (ECCO) and the Dutch guidelines, surveillance programs are recommended to start following 8 to 10 years of disease duration in children with UC, dictated by several risk factors (7,8). These surveillance colonoscopies should be performed with chromoendoscopy by an experienced pediatric or adult gastrointestinal endoscopist, based on ECCO-ESPGHAN guidelines (7,9). The Dutch guideline, however, lacks recommendations on these practical items (8). In addition, whereas the ECCO-ESPGHAN guideline recommends performing a screening colonoscopy 10 years after disease onset in all patients with UC (7–9), the Dutch guideline only mentions screening children with pancolitis (8). In both guidelines (7–9), the interval of surveillance after screening colonoscopy has not been well defined. Due to the paucity of data on surveillance in children with UC, all recommendations on surveillance colonoscopy for CRC in pediatric UC guidelines are based on data from adults.

The differences between guidelines, in combination with the rarity of dysplasia and CRC in pediatric UC, hypothetically result in different surveillance practices of Dutch pediatric gastroenterologists. This study aimed to evaluate the Dutch clinical practice regarding endoscopic surveillance for colorectal dysplasia and cancer in children with UC.

## METHODS

A clinical vignette-based survey was developed by the study investigators (PvR, NdB, TdM, DvS, JJ), which consisted of 13 multiple-choice and 11 open-ended questions (Supplementary Digital Material, http://links.lww.com/PG9/A129). This survey included respondents’ characteristics, adherence to guidelines, the performance of surveillance dictated by risk factors for CRC, three clinical vignettes (10), and practical aspects such as the endoscopy technique and by who the surveillance colonoscopy is conducted. Target respondents were pediatric gastroenterologists working in Dutch hospitals who treat children with UC. A paper version of the questionnaire was distributed at a section meeting for pediatric gastroenterology in the Netherlands on June 14, 2022. Pediatric gastroenterologists who did not attend the meeting, were sent the questionnaire (online version) by e-mail. All responses were analyzed in a descriptive manner and compared with the recommendations from the Dutch and European UC guidelines (Table 1) (7–9,11,12).

**TABLE 1.** Guidelines and corresponding recommendations

Guideline	Patients included in surveillance	First surveillance	Surveillance interval	Risk factors accounted for	Surveillance in patients with UC and PSC	Colonoscopy technique
NVK-guideline for IBD in children	Pediatric patients with ulcerative pancolitis	8–10 years after disease onset	–	–	–	–
ECCO-ESPGHAN guideline for pediatric UC	All pediatric patients with UC	Dictating for risk factors: 8–10 years after disease onset	–	- Disease extent - Disease severity - Family history of CRC - PSC	When ≥12 years old: annual or biannual	1. Chromoendoscopy 2. High-definition endoscopy with random and targeted biopsies
Endoscopy in Pediatric Inflammatory Bowel Disease: A Position Paper on Behalf of the Porto IBD Group	All pediatric patients with UC	10 years after disease onset or 8 years if >16 years old with risk factors	1, 3 or 5 years, based on present risk factors	- Disease extent - Disease severity - First degree relative with CRC <50 years - PSC	When ≥12 years old: annual or biannual	1. High-definition and/or chromoendoscopy 2. Conventional endoscopy with random biopsies
NVMDL guideline for UC in adults	Patients with UC, excluding patients with ulcerative proctitis	8 years after disease onset	1, 3 or 5 years, based on present risk factors	- Disease extent - First degree relative with CRC - PSC - Postinflammatory polyps - Stricture - Dysplasia <5 years	Annual	1. Chromoendoscopy 2. White-light endoscopy with targeted biopsies
ECCO-ESGAR Guideline for Diagnostic Assessment in IBD	Patients with UC, excluding patients with ulcerative proctitis	8 years after disease onset	1 to 5 years, based on present risk factors	- Disease extent - Disease severity - First degree relative with CRC - PSC - Postinflammatory polyps - Stricture or dysplasia <5 years	Annual	1. Chromoendoscopy 2. White-light endoscopy with random and targeted biopsies

CRC = colorectal cancer, ECCO = European Crohn’s and Colitis Organization, ESGAR = European Society of Gastrointestinal and Abdominal Radiology, ESPGHAN = European Society of Pediatric Gastroenterology, Hepatology and Nutrition, IBD = inflammatory bowel disease, NVK = Dutch association of pediatrics, NVMDL = Dutch association of gastroenterology, PSC = primary sclerosing cholangitis, UC = ulcerative colitis

## RESULTS

### Respondents’ Characteristics

Among 47 pediatric gastroenterologists who are registered and working in the Netherlands, 33 filled in the questionnaire (response rate 70%): 27 respondents during the section meeting for pediatric gastroenterology and six respondents returned the questionnaire via e-mail. The respondents’ characteristics are depicted in Table 2. Twenty-nine of 33 respondents (88%) do perform endoscopic procedures in children.

**TABLE 2.** Respondents’ characteristics

Characteristics	Total	University hospital	General hospital
Number of respondents (%)	33 (100)	21 (64)	12 (36)
Median number of years of experience as a pediatric gastroenterologist (range)	12 (0–23)	14 (0–23)	8 (0–18)
Median number of children with UC treated by respondents at the moment of participation (range)	25 (3–150)^†^	40 (3–150)^†^	20 (10–50)
Number of respondents performing endoscopic procedures (%)	29 (91)*	18 (90)*	11 (92)
Number of respondents conducting endoscopic surveillance on their patients with pediatric UC (%)	23 (72)*	20 (100)*	3 (25)

UC = ulcerative colitis.

Of the 33 respondents, three were working in a fellowship of pediatric gastroenterology at a university hospital.

* Missing data: one respondent did not complete this question.

^†^ Missing data: two respondents did not complete this question.

### Clinical Practice of Surveillance

Twenty-three respondents (70%) conduct endoscopic surveillance in children with UC. Of the 10 respondents who do not perform surveillance colonoscopy, nine work in a general hospital, whereas one works in a university hospital. The respondents who do not conduct surveillance, substantiated this with the argument that all their patients with UC had a disease onset >10 years old and thus do not enter the surveillance program before transition to a gastroenterologist.

#### Adherence to Guidelines

**FIGURE 1.**
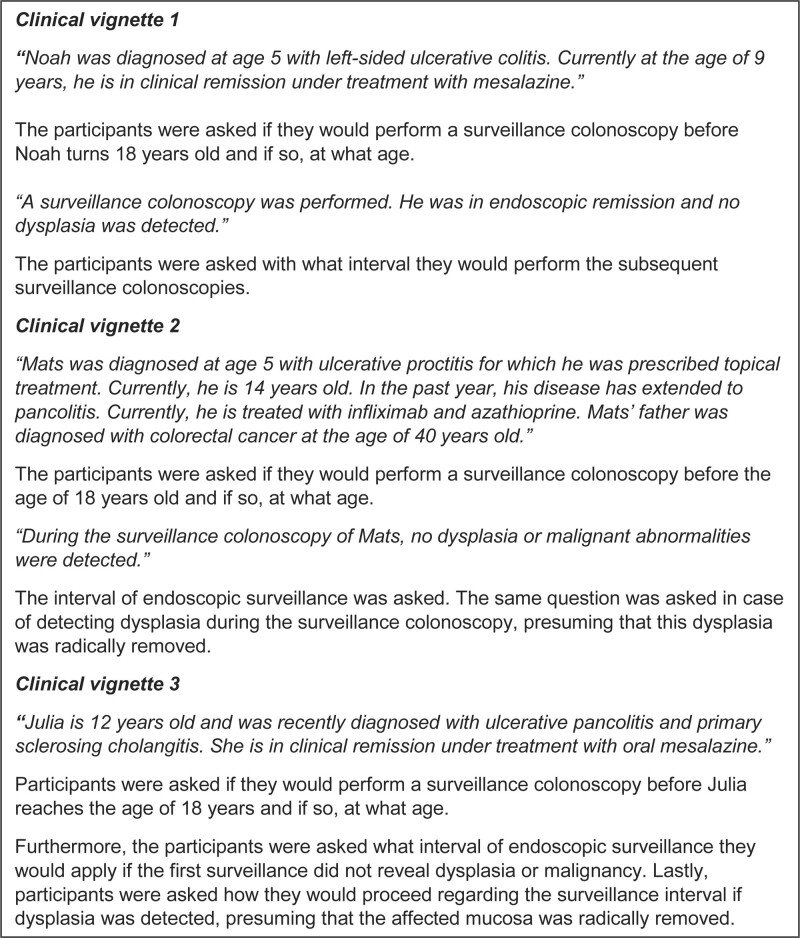
Clinical vignettes.

Twenty-seven respondents (82%) adhered to one or more available guidelines on surveillance colonoscopy in pediatric UC, while six respondents (18%) did not. The reported guidelines were: ECCO-ESPGHAN guidelines (n = 21) (7,13), the position paper “endoscopy in pediatric IBD” by the Porto IBD group (n = 1) (9), the Dutch guideline for pediatric UC (n = 5) (8) and UC guidelines in adults (n = 7) (11,12) (Supplemental Digital Figure 1, http://links.lww.com/PG9/A130). Twenty-four of 31 respondents (77%) indicated the need for the development of a new guideline, incorporating risk factors and extent of disease activity.

#### Risk Factors for Colorectal Cancer Guiding Decision-making for Surveillance Interval

The most frequently reported risk factors for CRC guiding decision-making for the surveillance interval were primary sclerosing cholangitis (PSC) (96% of respondents), dysplasia <5 years (89% of respondents), first degree family member with CRC <50 years old (85% of respondents), and chronic active disease (86% of respondents). Regarding disease extent, pancolitis would be taken into account as a risk factor by 52% of respondents (Supplemental Digital Table 1, http://links.lww.com/PG9/A131). Whereas PSC, a first degree relative with CRC, and disease extent were stated as risk factors in all available guidelines (7,9,11,12), dysplasia was only included in the adult guidelines (11,12). Disease severity was included in the guidelines, with the exception of the Dutch adult UC guideline (11). Although the use of immunomodulators is not included as a risk factor in the available guidelines, three respondents would take thiopurine use into account regarding the timing of surveillance. Variation in clinical practice was witnessed concerning children with ulcerative proctitis: 9/27 respondents (33%) would perform endoscopic surveillance regardless of the presence of risk factors, 5/27 respondents (19%) only if one or more risk factors were present, and 10 respondents (37%) would not perform endoscopic surveillance in these specific cases. Patients with ulcerative proctitis are excluded from the surveillance program according to the Dutch pediatric UC guideline (8) and the adult IBD guidelines (11,12), while the European pediatric UC guidelines include all children with UC in their recommendations (7,9,13).

### Clinical Vignettes

#### Responses Vignette 1

In case 1 (Figure 1), 30 out of 33 respondents (91%) would perform the first endoscopic surveillance <18 years old. Twenty-one of these respondents (70%) would perform the first surveillance 8–10 years after diagnosis: 14 respondents (48%) 8 years after diagnosis, and 5 respondents (17%) 10 years after diagnosis. Responses ranged between 10 and 17.5 years old at first surveillance. All available guidelines recommend performing the first surveillance 8–10 years after disease onset (7–9,11,12). However, in the absence of risk factors, as in this clinical vignette, the first surveillance could be performed after 10 years following disease onset (consistency rate 17%) (7,9). Ten out of 30 respondents (33%) would perform surveillance with an interval of 5 years, as recommended for those without any risk factors according to guidelines (9,11,12). Other intervals ranged between 1 and 10 years.

#### Responses Vignette 2

In case 2, 29 out of 32 respondents (91%) would perform surveillance <18 years old, of whom 22 (76%) respondents after 8–10 years following diagnosis. According to the ECCO-ESPGHAN guideline (7) and the Porto IBD group position paper (9), the first surveillance could be considered 8–10 years after disease onset, dictated by risk factors, as a first degree relative with CRC in this vignette. The other guidelines (8,11,12), however, exclude patients with ulcerative proctitis from the surveillance program. Hence, the first surveillance should be performed 8 years after the disease has extended to a pancolitis (>18 years of age).

If no dysplasia was detected, 9 out of 31 respondents (29%) applied a surveillance interval of 5 years, and three respondents (10%) an interval of 3 years. Other intervals ranged from 1 to 25 years. Five respondents (16%) would perform the subsequent surveillance just before transition to a gastroenterologist (18 years). While the ECCO-ESPGHAN and Dutch pediatric UC guidelines lack recommendations on the surveillance interval, the adult UC guidelines (11,12) recommend performing ongoing surveillance 3 years after screening surveillance in case of a first degree relative with CRC (consistency rate 10%). In case of radically removed dysplasia, 13 out of 31 respondents (42%) would use an interval of 1 year as recommended by the adult UC guidelines (11,12), and 6 respondents (19%) would use an interval of 2 years. Other responses ranged from 6 months to 5 years. Furthermore, six respondents (19%) indicated that they did not know how to proceed after detecting dysplasia.

#### Responses Vignette 3

**FIGURE 2.**
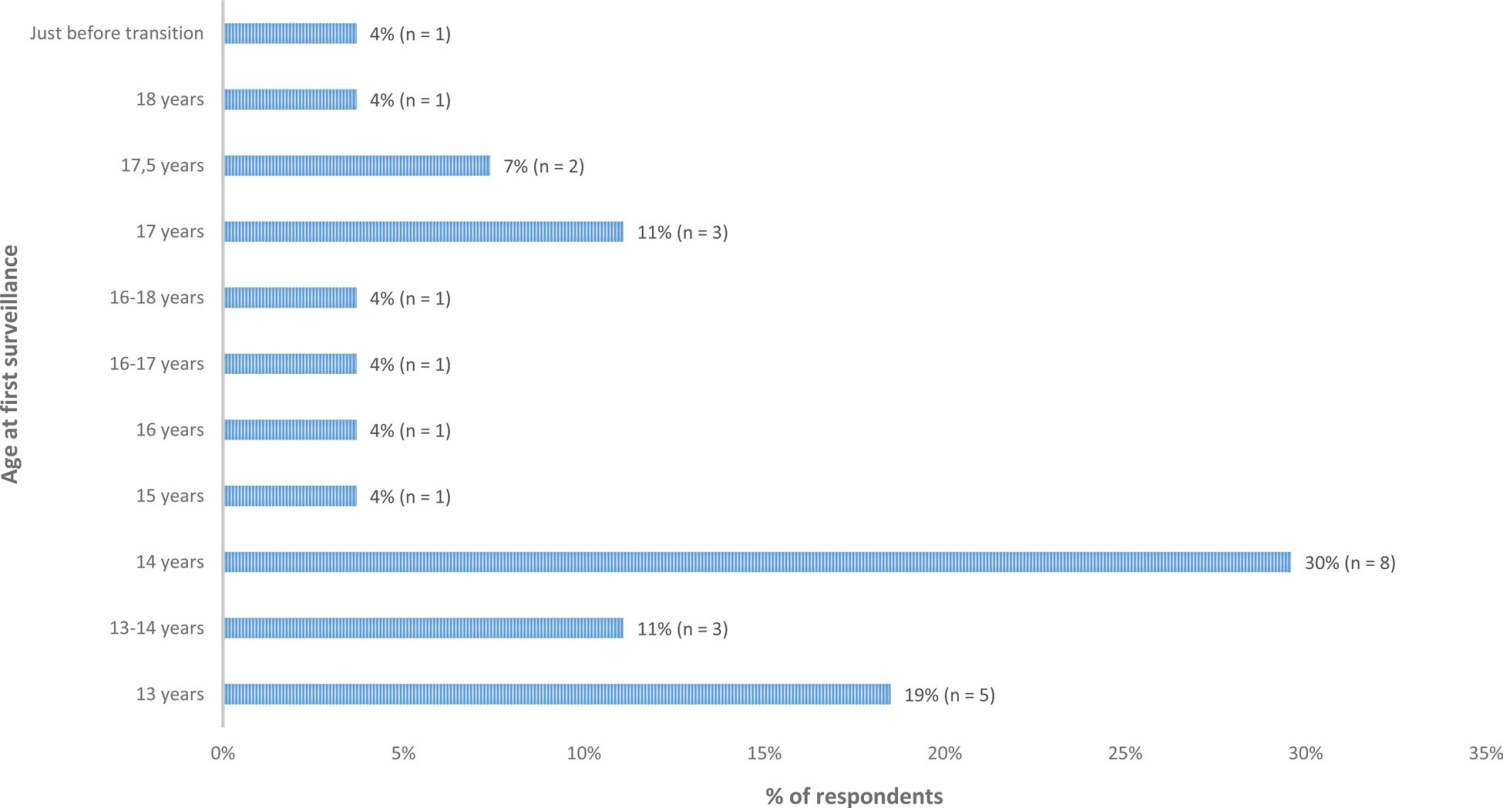
The variation in ages at the first endoscopic surveillance, reported by the respondents in case 3 of the clinical vignettes. Twenty-seven respondents completed this question.

In this case, endoscopic surveillance before 18 years old would be performed by 27 out of 32 respondents (84%). The ages at which the first surveillance would be performed by the respondents ranged from 13 to 18 years old (Figure 2). According to the available guidelines (7–9,11,12), annual or at least bi-annual surveillance colonoscopy should be initiated from the time of PSC diagnosis. Sixteen of 32 respondents would perform the screening colonoscopy after 1–2 years (consistency rate 50%).

If no dysplasia was detected by surveillance colonoscopy, 2 out of 28 respondents would use a surveillance interval of 1 year (7%), 6 respondents of 1–2 years (21%), and 9 respondents of 2 years (32%). Other intervals varied from 1 to 5 years. In case of radically removed dysplasia, 16 out of 28 respondents (57%) would perform surveillance annually. Other intervals pursued were as follows: every 6 months, every 2 years, and the first after half a year and thereafter yearly. Seven respondents (25%) did not know how to proceed in such a case and would consult a gastroenterologist. In both cases (dysplasia or no dysplasia), ongoing surveillance is recommended with an interval of 1 year according to the guidelines (9,11,12).

### Surveillance Colonoscopy Techniques

Chromoendoscopy and high-definition endoscopy, as recommended in the available guidelines (7–9,11,12), were used by only 2/24 respondents (8%) and 6/24 respondents (25%), respectively. Fourteen respondents (58%), of whom 13 work in a university hospital and one in a general hospital, performed surveillance by using conventional endoscopy with random biopsies (n = 12) or targeted biopsies (n = 2), mainly due to the availability and their experience in conducting this technique (Table 3).

**TABLE 3.** Colonoscopy techniques used by the respondents and corresponding arguments for their decision

Used colonoscopy technique	Number of respondents (%)*	Corresponding arguments, *n*
Chromoendoscopy with targeted biopsies and polypectomy or high-definition endoscopy with random biopsies^†^	2 (8)	- Depending on availability of gastroenterologist (n = 2)
High-definition endoscopy with random biopsies (for instance: every 10 cm 4 quadrants)	2 (8)	- According to practice of gastroenterologist (n = 2)
High-definition endoscopy with biopsies of visible lesions and, if necessary, polypectomy	4 (17)	- Following practice of gastroenterologist (n = 2) - Unclear added value of chromoendoscopy (n = 1) - Unclear added value of random biopsies (n = 1) - Experience with the technique (n = 2)
Conventional endoscopy with random biopsies, biopsies of visible lesions and, if necessary, polypectomy	12 (50)	- Experience with the technique (n = 5) - Logistically convenient (n = 1) - Availability (n = 3)
Conventional endoscopy with biopsies of visible lesions and, if necessary, polypectomy	2 (8)	- Appears sufficient (n = 2) - Logistically convenient (n = 1)
Other technique: -Endoscopy; if abnormal, consulting a gastroenterologist -In agreement with a gastroenterologist	1 (4) 1 (4)	n.a.

* Twenty-four participants completed the question. Nineteen participants outlined one or more arguments for their decision.

^†^ Two participants used two different techniques: chromoendoscopy when performing surveillance together with a gastroenterologist and high-definition endoscopy with random biopsies when performing surveillance alone.

### Who Conducts the Surveillance Colonoscopy

Fourteen of 27 respondents (52%) would perform the surveillance colonoscopy by themselves, while six respondents (22%) would consult a colleague pediatric gastroenterologist to perform the surveillance. The expertise of a gastroenterologist is requested or perceived as necessary by 7 respondents (26%). One participant specifically reported that the surveillance is now performed by himself or herself but that he or she feels that a gastroenterologist should perform the surveillance colonoscopy to obtain optimal quality.

#### Practical or Logistical Challenges

Nineteen respondents reported practical or logistical problems regarding the performance of endoscopic surveillance. This included the lack of experience/expertise in conducting surveillance colonoscopy, which was mentioned by 10 of 33 respondents (30%). Eight of 19 respondents (42%) reported that they experienced logistical problems in planning the surveillance colonoscopy, specifically when the expertise of a gastroenterologist is requested (n = 5).

### Outcomes of Surveillance

None of the respondents who performed surveillance have detected dysplasia or CRC in their patients with UC so far.

## DISCUSSION

This is the first survey study evaluating the variation in clinical practice on surveillance colonoscopy for CRC in pediatric UC among pediatric gastroenterologists. Around 70% of respondents performed endoscopic surveillance in children with UC. The remaining respondents specifically stated that their UC patients did not enter the surveillance program due to an older age of disease onset, which illustrated the high awareness of CRC screening in pediatric UC among pediatric gastroenterologists. Approximately 82% of respondents adhered to one or more guidelines on surveillance for CRC in children with UC, mainly the ECCO-ESPGHAN guidelines (7,13). Nevertheless, large variation in clinical practice was observed, especially in the surveillance colonoscopy technique, the surveillance intervals and taking the disease extent into account.

### Start Surveillance and Intervals Based on Risk Factors for Colorectal Cancer

According to the ECCO-ESPGHAN guideline (7), endoscopic surveillance should start 8–10 years after disease onset, dictated by risk factors. In *clinical vignette 1* of this study, no risk factors for CRC were present. However, 14 respondents would perform surveillance 8 years after diagnosis as compared to 5 respondents who started 10 years after diagnosis. This showed that a subset of pediatric gastroenterologists start CRC screening earlier in pediatric UC than recommended. Furthermore, these clinical vignettes showed large variation in clinical practice regarding the surveillance intervals after the first screening for CRC has been performed. This variation could be attributed to the limited experience of surveillance intervals among pediatric gastroenterologists. Additionally, recommendations on surveillance intervals are lacking in the ECCO-ESPGHAN and national guidelines (7,8).

Variation in surveillance practice was also observed regarding the disease extent in children with UC. Extensive disease is a well-known risk factor for CRC (2), as stated in the UC guidelines for children and adults. In the adult UC guidelines (11,12), patients with ulcerative proctitis are excluded from the surveillance program. This recommendation is based on research providing evidence that the risk for dysplasia and CRC in these patients is not higher than the risk in the general population (14). In the ECCO-ESPGHAN guideline (7), however, surveillance is recommended for all children with UC after 10 years of disease onset. These differences between guidelines have probably resulted in the varying surveillance practices of Dutch pediatric gastroenterologists in children with proctitis. Data on the risk of CRC in children with ulcerative proctitis is lacking, which hampers the development of evidence-based recommendations. Overall, it remains largely unknown to what extent factors such as disease localization and a family history of CRC influence the risk of developing dysplasia or CRC in pediatric patients with UC. Therefore, future research is recommended on using these risk factors in early surveillance programs in UC patients <18 years of age.

A concomitant diagnosis of PSC has also been recognized as a firm independent risk factor for dysplasia and CRC (7,9,11,12). This has been investigated in a multicenter study including 509 children with UC and PSC (15). The findings of this study showed that one patient developed CRC <18 years old after 10 years from diagnosis and 4 patients developed colonic dysplasia <18 years (3 months, 1, 4, and 10 years after diagnosis) (15). The UC guidelines in children and adults (7,9,11,12) recommend to conduct annual or biannual surveillance colonoscopy from the time of PSC diagnosis. The responses of *clinical vignette 3* in the present study showed that 84% of respondents would perform surveillance in a girl who was diagnosed at age 12 with pancolitis and PSC. Of these respondents, 59% would conduct surveillance within 2 years from the diagnosis of PSC. Hence, there was discrepancy between clinical practice and the guideline recommendations on surveillance in this case. This indicates that education of Dutch pediatric gastroenterologists concerning the importance of surveillance for CRC in children with UC and PSC is needed.

### Surveillance Colonoscopy Techniques

There was a large inconsistency between the applied surveillance techniques by the Dutch pediatric gastroenterologists and the recommendations in all available UC guidelines in children and adults with UC (7,9,11,12). According to these guidelines, chromoendoscopy is the recommended technique for surveillance. However, this technique was used by only two of 24 respondents (8%). The guidelines differ in their recommendation for the second choice technique. The European guidelines recommend to use high-definition or white-light endoscopy with random biopsies as second choice technique (7,11), whereas the Dutch guideline for IBD in adults recommends to perform white-light endoscopy with targeted biopsies (12). Most respondents in the present study, however, performed conventional endoscopy with harvesting of random biopsies, mainly based on availability and experience with this technique.

Ten respondents (30%) mentioned a lack of expertise/experience in conducting surveillance colonoscopy, while 7 of them conduct the endoscopic surveillance by themselves. A quarter of respondents who perform surveillance would request the expertise of a gastroenterologist. However, multiple respondents experienced logistic problems regarding the planning and performance of the surveillance in collaboration with an experienced gastroenterologist. These responses highlight the need for an interprofessional collaboration and strict arrangements with regard to the performance of surveillance in children.

### Outcomes of Surveillance for Colorectal Cancer

None of the respondents who performed surveillance have ever detected dysplasia or CRC in their patients with UC. Although pediatric UC is associated with an increased risk of developing CRC later in life (1,2,14,15), cases of CRC related to UC in childhood are extremely rare (3,4,16–18). These data challenge the current guidelines recommending patients with UC to undergo surveillance even when aged <18 years old. Since randomized controlled trials on the benefits and costs of surveillance practices in pediatric UC are unethical, future research based on population-based cancer registries from multiple countries is recommended to study the risk of CRC in children with UC.

### Strengths and Limitations

The strength of this study was that the questionnaire was distributed during an in-person section meeting for pediatric gastroenterology in the Netherlands, thereby reducing the risk of selection bias. A limitation was the moderate response rate of 70% and thereby the limited sample size of 33 respondents. This clinical vignette-based survey was limited to the surveillance practices of pediatric gastroenterologist in the Netherlands, since adherence to both national and European guidelines was assessed. However, this has led to a limited generalizability. No standardized questionnaire was available to evaluate the practice of endoscopic surveillance in pediatric UC. Furthermore, two different study settings (paper and online version of the questionnaire) were used. During the in-person section meeting, there was a time limit to complete the questionnaire which prevented respondents from extensively studying available guidelines. For that reason, the obtained results might deviate from real-life practice. The respondents who returned the questionnaire by mail, on contrary, did not have a time limit. This could have led to more heterogeneity in the responses obtained.

## CONCLUSION

In conclusion, the need for endoscopic surveillance programs for dysplasia and CRC in pediatric UC patients were universally recognized by Dutch pediatric gastroenterologists. However, heterogeneity in clinical surveillance practices was observed, particularly regarding the initiation and interval of surveillance, and the applied colonoscopy techniques. Additionally, the responses underlined the need for a close collaboration with gastroenterologists. The benefits of conducting dysplasia and CRC screening in children with UC remains debatable due to the limited available data on this topic. Therefore, future research on the incidence of dysplasia or CRC development in children with UC <18 years old is needed, including the assessment of risk factors and optimal endoscopic technologies. These data could aid in the development of a (more) evidence-based guidelines regarding surveillance in pediatric UC.

## Supplementary Material

## References

[1] Olen O Askling J Sachs MC. Childhood onset inflammatory bowel disease and risk of cancer: a Swedish nationwide cohort study 1964-2014. BMJ. 2017;358:j3951. 28931512 10.1136/bmj.j3951 PMC5605779

[2] Everhov AH Ludvigsson JF Järås J. Colorectal cancer in childhood-onset inflammatory bowel disease: a scandinavian register-based cohort study, 1969-2017. J Pediatr Gastroenterol Nutr. 2022;75:480–484. 36125530 10.1097/MPG.0000000000003574

[3] de Ridder L Turner D Wilson DC; Porto IBD Working Group of ESPGHAN. Malignancy and mortality in pediatric patients with inflammatory bowel disease: a multinational study from the porto pediatric IBD group. Inflamm Bowel Dis. 2014;20:291–300. 24374875 10.1097/01.MIB.0000439066.69340.3c

[4] Abraham BP Mehta S El-Serag HB. Natural history of pediatric-onset inflammatory bowel disease: a systematic review. J Clin Gastroenterol. 2012;46:581–589. 22772738 10.1097/MCG.0b013e318247c32f PMC3972042

[5] Lindberg J Stenling R Palmqvist R. Early onset of ulcerative colitis: long-term follow-up with special reference to colorectal cancer and primary sclerosing cholangitis. J Pediatr Gastroenterol Nutr. 2008;46:534–538. 18493208 10.1097/MPG.0b013e31815a98ef

[6] Lutgens MW Vleggaar FP Schipper ME. High frequency of early colorectal cancer in inflammatory bowel disease. Gut. 2008;57:1246–1251. 18337322 10.1136/gut.2007.143453

[7] Turner D Ruemmele FM Orlanski-Meyer E. Management of paediatric ulcerative colitis, part 1: ambulatory care-an evidence-based guideline from european crohn’s and colitis organization and european society of paediatric gastroenterology, hepatology and nutrition. J Pediatr Gastroenterol Nutr. 2018;67:257–291. 30044357 10.1097/MPG.0000000000002035

[8] Escher JC Aardoom MA Van den Brink G Diederen K Van Gaalen MAC. Richtlijn-Update Inflammatoire Darmziekten (Ibd) Bij Kinderen En Adolescenten. Van Zuiden Communications B.V; 2018.

[9] Oliva S Thomson M de Ridder L. Endoscopy in pediatric inflammatory bowel disease: a position paper on behalf of the porto IBD Group of the European society for pediatric gastroenterology, hepatology and nutrition. J Pediatr Gastroenterol Nutr. 2018;67:414–430. 30130311 10.1097/MPG.0000000000002092

[10] Peabody JW Luck J Glassman P. Comparison of vignettes, standardized patients, and chart abstraction: a prospective validation study of 3 methods for measuring quality. JAMA. 2000;283:1715–1722. 10755498 10.1001/jama.283.13.1715

[11] Maaser C Sturm A Vavricka SR; European Crohn’s and Colitis Organisation [ECCO] and the European Society of Gastrointestinal and Abdominal Radiology [ESGAR]. ECCO-ESGAR guideline for diagnostic assessment in IBD Part 1: Initial diagnosis, monitoring of known IBD, detection of complications. J Crohns Colitis. 2019;13:144–164. 30137275 10.1093/ecco-jcc/jjy113

[12] van Asseldonk DP van Bodegraven AA de Boer KHN. HANDLEIDING BEHANDELING IBD – 2014-2015 - Moderniseren van de Richtlijn IBD 2009. initiative on Crohn’s and Colitis. ICC; 2015. 69–74.

[13] Turner D Ruemmele FM Orlanski-Meyer E. Management of paediatric ulcerative colitis, part 2: acute severe colitis-an evidence-based consensus guideline from the european crohn’s and colitis organization and the european society of paediatric gastroenterology, hepatology and nutrition. J Pediatr Gastroenterol Nutr. 2018;67:292–310. 30044358 10.1097/MPG.0000000000002036

[14] Lutgens MW van Oijen MG van der Heijden GJ. Declining risk of colorectal cancer in inflammatory bowel disease: an updated meta-analysis of population-based cohort studies. Inflamm Bowel Dis. 2013;19:789–799. 23448792 10.1097/MIB.0b013e31828029c0

[15] El-Matary W Guthery SL Amir AZ. Colorectal dysplasia and cancer in pediatric-onset ulcerative colitis associated with primary sclerosing cholangitis. Clin Gastroenterol Hepatol. 2021;19:1067–1070.e2. 32360820 10.1016/j.cgh.2020.04.055 PMC8788582

[16] Kim MJ Ko JS Shin M. Colorectal cancer associated with pediatric inflammatory bowel disease: a case series. BMC Pediatr. 2021;21:504. 34763671 10.1186/s12887-021-02966-9 PMC8582128

[17] Jang J Lee SH Jeong IS. Clinical characteristics and long-term outcomes of pediatric ulcerative colitis: a single-center experience in Korea. Gut Liver. 2022;16:236–245. 34238767 10.5009/gnl20337 PMC8924810

[18] Atia O Harel S Ledderman N. Risk of cancer in paediatric onset inflammatory bowel diseases: a nation-wide study from the epi-IIRN. J Crohns Colitis. 2022;16:786–795. 34791097 10.1093/ecco-jcc/jjab205

