# Supplementary material for: Endoscopic Surveillance for Colorectal Cancer in Pediatric Ulcerative Colitis: A Survey Among Dutch Pediatric Gastroenterologists
Source: JPGN Rep. 2023 Jul 17;4(3):e341. doi: 10.1097/PG9.0000000000000341 (PMC10435030; doi:10.1097/PG9.0000000000000341)
Supplement: Supplementary file 1 [file pg9-4-e341-s001.pdf]

Supplementary material

Survey:
Endoscopic surveillance for colorectal dysplasia and cancer in children with ulcerative colitis

Through this clinical vignette-based questionnaire, we aim to evaluate the Dutch clinical practice by pediatricians with regard to endoscopic surveillance in children with ulcerative colitis.

It takes approximately 10 minutes to fill in the questionnaire.

Thank you in advance for your participation.

For questions you can e-mail to d.vanschie2@amsterdamumc.nl.

On behalf of the KiCC (Kids with Crohn's and colitis):

D.A. van Schie, sixth year medical student
J.Z. Jagt, medical doctor / PhD student
Dr. N.K.H. de Boer, gastroenterologist
Dr. P.F. van Rheenen, pediatric gastroenterologist
Dr. T.G.J. de Meij, pediatric gastroenterologist

Section I

General questions

1. For how many years are you working as a pediatric gastroenterologist?

   ……………

2. How many children with ulcerative colitis are you currently treating?

   ……………

3. In what type of hospital are you working?

   ☐ General ☐ University

4. Do you perform endoscopic procedures on children?

   ☐ Yes ☐ No

Section II

Clinical cases with regard to surveillance colonoscopy in children with ulcerative colitis.

Case 1: Noah was diagnosed at age 5 with left-sided ulcerative colitis. Currently at the age of 9 years, he is in clinical remission under treatment with mesalazine.

5. Would you (let a colleague) perform a surveillance colonoscopy on Noah before he turns 18 years old?

   ☐ Yes (move on to question 6) ☐ No (move on to case 2)

6. A.) At what age would you (let a colleague) perform the first surveillance colonoscopy?

   ……………

   "A surveillance colonoscopy was performed. He was in endoscopic remission and no dysplasia was detected."

   B.) What surveillance interval do you choose afterward?

   ……………

7. How do you take into account the disease activity in your decision with regard to the timing of a surveillance colonoscopy?

   ……………

Case 2: Mats was diagnosed at age 5 with ulcerative proctitis for which he was prescribed topical treatment. Currently, he is 14 years old. In the past year, his disease has extended to a pancolitis. Now, he is treated with infliximab and azathioprine. Mats' father was diagnosed with colon cancer at the age of 40 years old.

8. Would you (let a colleague) perform a surveillance colonoscopy on Mats before he turns 18 years old?

   ☐ Yes (move on to question 9) ☐ No (move on to case 3)

9. At what age would you (let a colleague) perform the first surveillance colonoscopy?

   ……………

During the first surveillance colonoscopy of Mats, no dysplasia or malignant abnormalities were detected.

10. After how many years will you, based on this information, let Mats undergo his subsequent surveillance colonoscopy?

    ……………

Suppose that you have detected dysplasia at Mats' surveillance colonoscopy, which was radically removed.

11. In this case, with what frequency would you perform colonoscopic surveillance on Mats?

    ……………

<u>Case 3</u>: Julia is 12 years old and was recently diagnosed with ulcerative pancolitis and primary sclerosing cholangitis. She is in clinical remission under treatment with oral mesalazine.

12. Would you (let a colleague) perform a surveillance colonoscopy on Julia before she turns 18 years old?
    - ☐ Yes (move on to question 13)
    - ☐ No (move on to section III)

13. A.) When would you (let a colleague) perform the first surveillance colonoscopy on Julia?

    ……………

    B.) In case no dysplasia or malignancy is detected, what surveillance interval would you choose?

    ……………

Suppose that you have detected dysplasia at Julia's surveillance colonoscopy, which was radically removed.

14. With what frequency would you perform colonoscopic surveillance on Julia?

    ……………

Section III

Indication of colonoscopic surveillance in children with ulcerative colitis.

15. Which guideline do you follow for deciding when and how colonoscopic surveillance for colorectal cancer would be performed in children with ulcerative colitis?
    - ☐ ECCO-ESPGHAN guideline: management of pediatric ulcerative colitis
    - ☐ NVK-guideline: IBD in children
    - ☐ Different, namely: ……………
    - ☐ NVMDL guideline for IBD in adults
    - ☐ Hospital guideline
    - ☐ I do not follow a specific guideline

16. Do you feel the need for a new guideline with regard to colonoscopic surveillance in children with ulcerative colitis?
    - ☐ Yes
    - ☐ No, satisfied with the current guideline

17. Do you, or a colleague (pediatric) gastroenterologist, ever perform a surveillance colonoscopy on <u>your</u> pediatric ulcerative colitis patients?
    - ☐ Yes
    - ☐ No, because: ……………

If you answered "no" on question 17, the questionnaire ends here.

18. Do you perform colonoscopic surveillance in children with ulcerative proctitis?
   - ☐ Yes
   - ☐ No
   - ☐ Yes, but only if one or more risk factors are present

19. Have you ever detected dysplasia or malignant abnormalities in one of your patients with pediatric ulcerative colitis through surveillance colonoscopy?
   - ☐ Yes, namely:

     ……………
   - ☐ No

20. What risk factors for developing colorectal cancer do you take into account with regard to the timing of a surveillance colonoscopy? More than one answer possible.
   - ☐ Primary sclerosing cholangitis
   - ☐ Stricture at previous colonoscopy
   - ☐ Dysplasia in the past 5 years
   - ☐ Presence of post inflammatory polyps
   - ☐ First degree family member with colorectal cancer <50 years
   - ☐ First degree family member with colorectal cancer >50 years
   - ☐ First degree family member with IBD
   - ☐ Usage of thiopurine
   - ☐ Chronic disease activity
   - ☐ Pancolitis
   - ☐ None of the above

Section IV

Used colonoscopy technique in surveillance for colorectal cancer in children with ulcerative colitis.

21. What colonoscopy technique is used on your pediatric patients in conducting surveillance?
   - ☐ Chromo endoscopy with targeted biopsies + polypectomy
   - ☐ High definition endoscopy with random biopsies (for example every 10 cm 4 quadrants)
   - ☐ High definition endoscopy with solely biopsies of visible lesions and, if necessary, polypectomy
   - ☐ Normal endoscopy with random biopsies + biopsies of visible lesions + polypectomy
   - ☐ Normal endoscopy with biopsies of visible lesions + polypectomy
   - ☐ Different, namely: ……………

22. Why do you choose this technique? …………….

23. Who performs the surveillance colonoscopy on your patients with ulcerative colitis?
   - ☐ I perform the procedure myself
   - ☐ A colleague pediatric gastroenterologist with more experience regarding surveillance performs the procedure
   - ☐ A gastroenterologist
   - ☐ Different, namely: …………….

24. What logistic or substantive problems do you experience in conducting surveillance colonoscopy?

    …………….
