# Supplementary material for: Endoscopic Surveillance for Colorectal Cancer in Pediatric Ulcerative Colitis: A Survey Among Dutch Pediatric Gastroenterologists
Source: JPGN Rep. 2023 Jul 17;4(3):e341. doi: 10.1097/PG9.0000000000000341 (PMC10435030; doi:10.1097/PG9.0000000000000341)
Supplement: Supplementary file 2 [file pg9-4-e341-s002.pdf]

Supplementary Figure 1. This pie chart shows the number of respondents who followed each clinical guideline. A total of 40 answers were given by 33 respondents: six respondents followed more than one guideline.

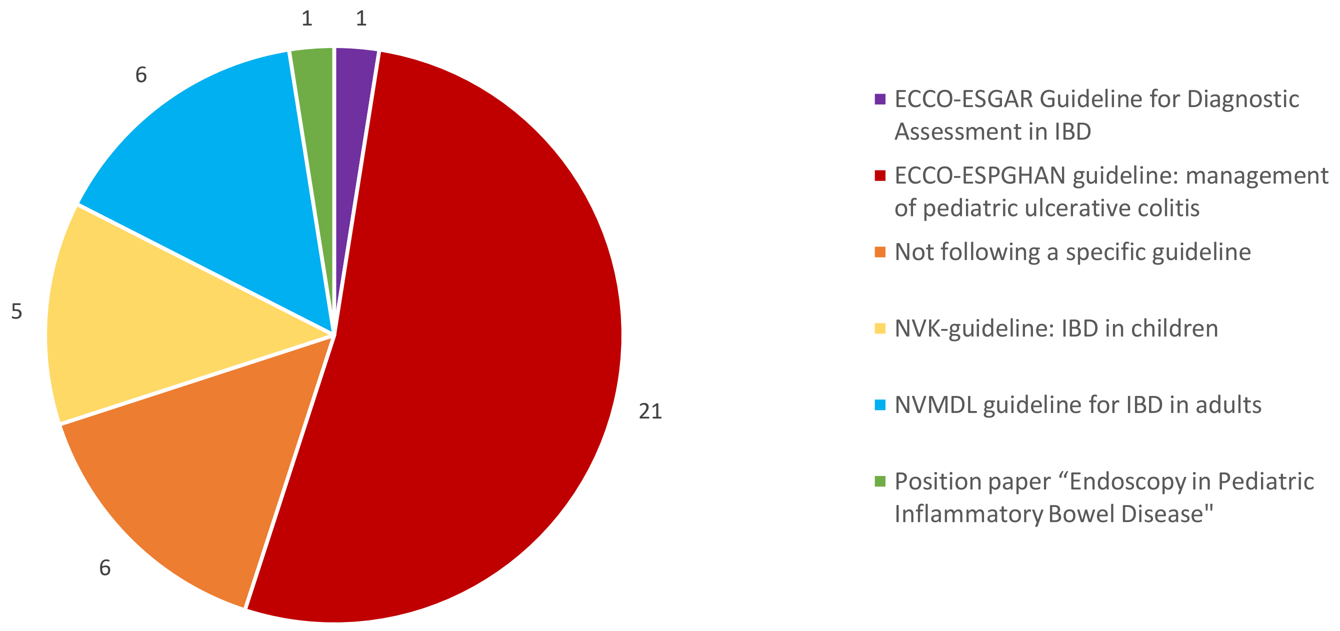

ECCO = European Crohn's and Colitis Organization, ESGAR = European Society of Gastrointestinal and Abdominal Radiology, ESPGHAN = European Society of Pediatric Gastroenterology, Hepatology and Nutrition, IBD = inflammatory bowel disease**,** NVK = Dutch association of pediatrics, NVMDL = Dutch association of gastroenterology
