# Supplementary material for: Endoscopic Surveillance for Colorectal Cancer in Pediatric Ulcerative Colitis: A Survey Among Dutch Pediatric Gastroenterologists
Source: JPGN Rep. 2023 Jul 17;4(3):e341. doi: 10.1097/PG9.0000000000000341 (PMC10435030; doi:10.1097/PG9.0000000000000341)
Supplement: Supplementary file 3 [file pg9-4-e341-s003.pdf]

Supplementary Table 1. Risk factors for colorectal cancer guiding decision to perform surveillance colonoscopy in children with ulcerative colitis

Risk factor	Number of respondents (%)
Primary sclerosing cholangitis	26 (96)
Stricture	4 (15)
Dysplasia <5 years	24 (89)
Post inflammatory polyps	8 (30)
First degree family member with CRC <50 years old	23 (85)
First degree family member with CRC >50 years old	8 (30)
Chronic disease activity	23 (85)
Pancolitis	14 (52)

Twenty-seven respondents completed this question.
